# Research on early-warning index of the spatial temperature field in concrete dams

**DOI:** 10.1186/s40064-016-3659-2

**Published:** 2016-11-14

**Authors:** Guang Yang, Chongshi Gu, Tengfei Bao, Zhenming Cui, Kan Kan

**Affiliations:** 1 State Key Laboratory of Hydrology-Water Resources and Hydraulic Engineering, Hohai University, No. 1 Xikang Road, Nanjing, 210098 China; 2 National Engineering Research Center of Water Resources Efficient Utilization and Engineering Safety, Hohai University, Nanjing, 210098 China; 3 College of Water-Conservancy and Hydropower, Hohai University, Nanjing, 210098 China

**Keywords:** Ward spatial clustering, Temperature entropy, Projection pursuit, Early-warning, Synergetics, Spatial temperature field

## Abstract

Warning indicators of the dam body’s temperature are required for the real-time monitoring of the service conditions of concrete dams to ensure safety and normal operations. Warnings theories are traditionally targeted at a single point which have limitations, and the scientific warning theories on global behavior of the temperature field are non-existent. In this paper, first, in 3D space, the behavior of temperature field has regional dissimilarity. Through the Ward spatial clustering method, the temperature field was divided into regions. Second, the degree of order and degree of disorder of the temperature monitoring points were defined by the probability method. Third, the weight values of monitoring points of each regions were explored via projection pursuit. Forth, a temperature entropy expression that can describe degree of order of the spatial temperature field in concrete dams was established. Fifth, the early-warning index of temperature entropy was set up according to the calculated sequential value of temperature entropy. Finally, project cases verified the feasibility of the proposed theories. The early-warning index of temperature entropy is conducive to the improvement of early-warning ability and safety management levels during the operation of high concrete dams.

## Background

Temperature variation is one of main safety monitoring items during the operation of concrete dams. A scientific and reasonable early-warning index (Wu 2003) of the dam body’s temperature has important significance in accurate hazard recognition and dam security protection. Temperature control is a key factor influencing whether the temperature cracks occur or not. In the process of operation, improper temperature control (Zhu 2014) will lead to dam cracking and even endanger the safety of dam bodies. Generally, the concrete dams belong to the mass concrete structure which has bad temperature conductivity, and they are often exposed to the outside, contacting with water, air and so on. All kinds of factors like temperature change outside, cement hydration heat and constraint stress may produce tensile stress. With the limit ability of concrete tensile strength, cracks tend to appear in the mass concrete structure. Combined with long-acting service characteristics of the concrete dams, advanced mechanical and mathematical theories are employed to scientifical theory for early-warning, as well as to conduct timely and effective determination of the safety status of dams. These activities are critical to perform safe operations and determine crucial topics in dam safety monitoring field research.

To date, the warning methods of dam body’s temperature have obtained a series of research achievements. Temperature early-warning index (Su et al. 2011, 2012; Zhou et al. 2012) is an important index to control cracks of concrete dam. When the current concrete temperature exceeds early-warning index of temperature, effective temperature control measures must be taken to control the concrete temperature. In 2004, Wang took some temperature measuring points as constraint conditions, using three-dimensional finite element analysis method to represent the characters of the dam’s temperature field (Wang 2004). Wu applied sensitivity analysis method to determine temperature field’s main impact factors (Wu and Song 2011). Combined with the situation of the practical concrete dam temperature control, Qu et al. applied expert evaluation and the theory of entropy weight to establish a multi-objective fuzzy mathematical model for the concrete dams (Qu et al. 2012). Although the above-mentioned theories and methods complement and improve traditional methods in solving difficult problems in dam safety, but traditional warnings theories are targeted at a single point which have limitations, and the scientific warning theories on the global behavior of the spatial temperature field are non-existent. Thus, a scientific and accurate early-warning index based on the spatial temperature field should be studied to improve the warning ability of the concrete dams.

In this paper, accordingly, the spatial temperature field at different elevations and positions have different behaviors, that is, the regional dissimilarity exist. Through Ward spatial clustering, the spatial temperature field was divided into regions. On this basis, an expression of temperature entropy was proposed based on the synergetics and entropy. This expression can comprehensively evaluate the overall variation of temperature field in concrete dams. With this expression, the sequential value and the early-warning index value of temperature entropy was analyzed and determined via the small probability method. Finally, project cases verified the feasibility of the proposed theories.

### Research on the partitioning method of the spatial temperature field

**Fig. 1:**
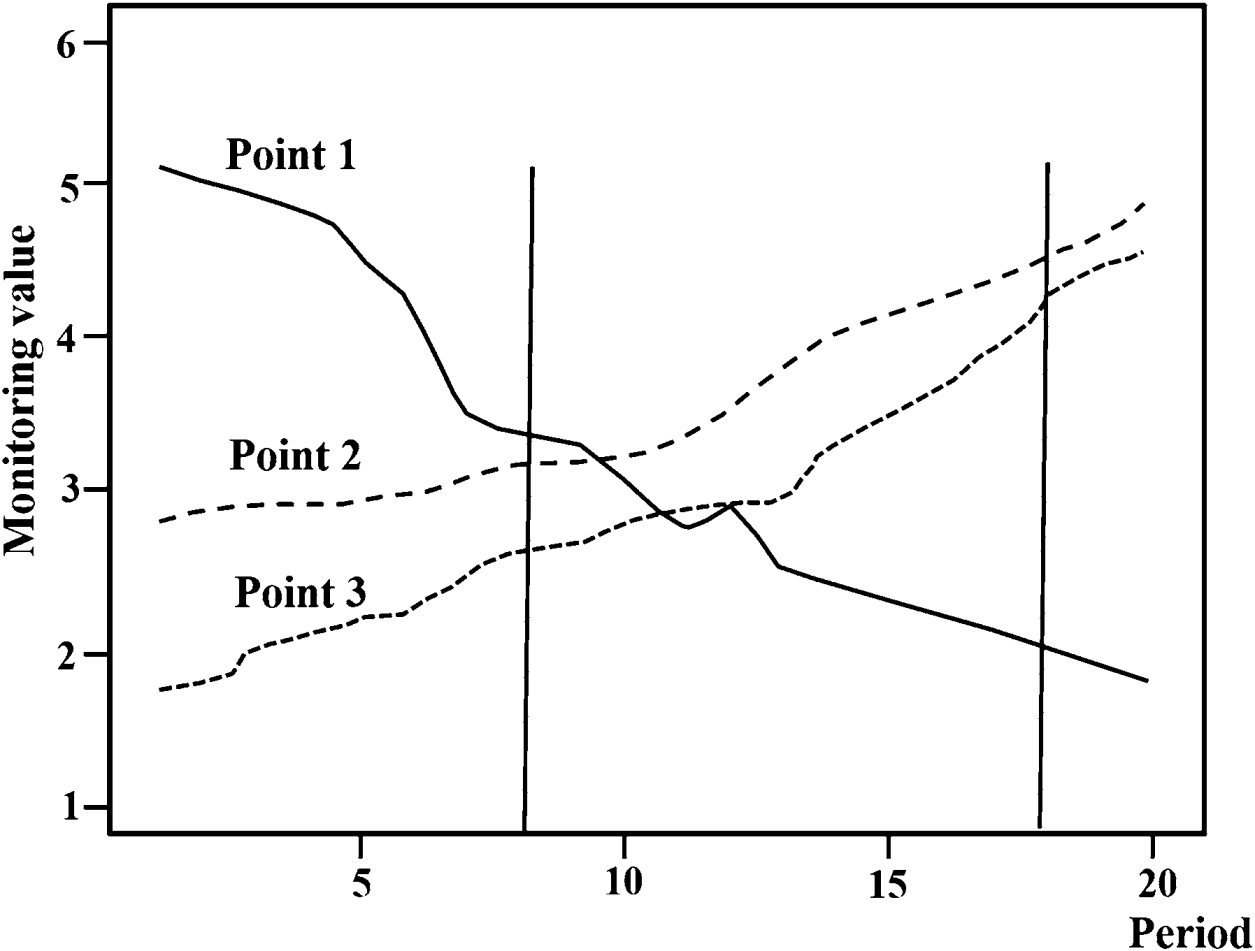
Development law of monitoring the measuring point values in different periods

The traditional partitioning method adopts the average value of the monitoring value time series. This method can only express the average variation condition of the monitoring values. Thus, information on the time dimension is considerably lost and an invisible hypothesis is generated, that is, the measuring points experience the same directional variation in the time dimension. Reflecting the real change rule of the structural behavior with time is difficult and unreasonable. The development law of the monitoring values of the measuring points in different periods is described in Fig. 1. If the traditional method is used, then measuring points 1 and 3 should be classified as one category. However, if the entire variation process is considered, then measuring points 2 and 3 should be classified as one category. If no hypothesis is formulated for the spatial temperature field, then partitioning is conducted according to the intimacy degree among all the observed values and based on Ward spatial clustering (Bertamini et al. 2016; Hu et al. 2016; Nascimento et al. 2011; Li et al. 2013). The partitioning principle attempts to make the similarity degree of the observed value change rule within the partitions considerably close while making the similarity degree of the observed value change rule between the partitions substantially low. The spatial temperature field partitioning needs to process two core problems: (1) selecting the type of statistical magnitude to characterize the similarity degree among the opening displacements of the measuring points and (2) selecting the criteria that should be used to determine the similarity between the partitions.

#### Measurement method of similarity degree among the monitoring values of the temperature measuring points

*N* is assumed as the number of all temperature monitoring points, *T* is the monitoring time series, and $x_{it} (i = 1,2, \ldots ,N;\, t = 1,2, \ldots ,T)$ represents the temperature data set. For set *x*_*it*_, *S*_*t*_ is the standard deviation at time *t*, and the following three distances are defined to describe the similarity degree among the monitoring values of the temperature measuring points.

The “absolute distance” between measuring points *m* and *n* is simplified as *d*_*mn*_(*AQED*) and is expressed as follows:

1 $$d_{mn} (AQED) = \left[ {\sum\limits_{t = 1}^{T} {(x_{mt} - x_{nt} )^{2} } } \right]^{1/2}$$

where $d_{mn} (AQED)$ characterizes the distance between measuring points *m* and *n* within the entire period.

The “incremental distance” between measuring points *m* and *n* is simplified as $d_{mn} (ISED)$ and is expressed as follows:

2 $$d_{mn} (ISED) = \left[ {\sum\limits_{t = 1}^{T} {\left( {\frac{{\Delta x_{mt} }}{{\Delta x_{mt - 1} }} - \frac{{\Delta x_{nt} }}{{\Delta x_{nt - 1} }}} \right)^{2} } } \right]^{1/2}$$

3 $$\Delta x_{mt} = x_{mt} - x_{mt - 1}$$

4 $$\Delta x_{nt} = x_{nt} - x_{nt - 1}$$

where $d_{mn} (ISED)$ depicts the difference in variation trends of the monitoring values of the temperature measuring points *m* and *n*. If the monitoring values presents same-directional change across time, then change is substantially coordinated, the two values will be similar, and $d_{mn} (ISED)$ will be considerably small. If the monitoring values presents reverse-directional change across time, then the similarity is poor and $d_{mn} (ISED)$ will be substantially high.

The “comprehensive distance” between measuring points *m* and *n* is simplified as $d_{mn} (CED)$ and is expressed as follows:

5 $$d_{mn} (CED) = \alpha d_{mn} (AQED) + \beta d_{mn} (ISED)$$

6 $$\alpha + \beta = 1 .$$

The “comprehensive distance,” which is a weighted array of “absolute distance” and “incremental distance,” comprehensively describes the similarity of change of the measured values at the measuring points.

#### Measurement method of similarity degree among the different spatial temperature field partitions

All of the measuring points are assumed to be divided into *k* partitions. These partitions are $G_{1} ,G_{2} , \ldots ,G_{k}$, *N*_*l*_ is the number of measuring points of the *G*_*l*_ category, $\bar{X}_{l}$ is the center of gravity of the measuring values of the *G*_*l*_ category, and *X*_*il*_ is the monitoring value of the *i*(*i* = 1, 2, …, *N*_*l*_) th measuring point in the *G*_*l*_ category. For the monitoring data of the *N* measuring points within *T* periods, the sum of the squares of deviations of the different measuring point series in the *G*_*l*_ partition of the dam is expressed as follows:

7 $$W_{l}^{ * } = \sum\limits_{i = 1}^{{N_{l} }} {\sum\limits_{t = 1}^{T} {\left[ {\omega_{1} (X_{it} - \overline{{X_{t} }} )^{{\prime }} (X_{it} - \overline{{X_{t} }} )} \right]} } + \sum\limits_{t = 2}^{T} {\left[ {\omega_{2} (Y_{it} - \overline{{Y_{t} }} )^{{\prime }} (Y_{it} - \overline{{Y_{t} }} )} \right]} .$$

The sum of the squares of deviations of the *k* partition is expressed as follows:

8 $$W^{ * } = \sum\limits_{i = 1}^{k} {W_{l}^{ * } }$$

where $W_{l}^{ * }$ is the sum of the squares of deviations of the *N*_*l*_ measuring values, *X*_*it*_ is the monitoring value of the measuring point *i* in period *t*, $Y_{it} = \frac{{\Delta X_{it} }}{{X_{it - 1} }}$ represents the relative increment of the monitoring values of the temperature measuring point *i* within the *t* period in *G*_*l*_, $\Delta X_{it} = X_{it} - X_{it - 1}$ represents the absolute quantity difference of the monitoring values of the temperature measuring point *i* in the *t* and *t* − 1 periods in *G*_*l*_, $\overline{{X_{t} }} = \frac{1}{N}\sum\nolimits_{t = 1}^{{N_{l} }} {X_{it} }$ and $\overline{{Y_{t} }} = \frac{1}{N}\sum\nolimits_{t = 1}^{{N_{l} }} {Y_{it} }$.

The assumption is that *n* combinations are conducted in the partitioning process based on the threshold value method, and the ratio of the distance between partitions in the *l*th partitioning to that in the last partitioning is $S_{l} = \frac{{D_{l} }}{{D_{n - 1} }}$. If the difference between *S*_*l*_ and *S*_*l*+1_ is small and that between *S*_*l*_ and *S*_*l*−1_ is large, then the corresponding distance *D*_*l*_ between the partitions can be the threshold value of the partition.

#### Partitioning flow

This study employed Ward spatial clustering as basis to propose a complete partitioning flow. The procedure involved is as follows.

- Step 1: Expressions (1) and (2) are used to calculate the “absolute distance” and “incremental distance,” respectively.
- Step 2: The calculated coefficient values are substituted into expression (5), and the comprehensive distance *d*_*mn*_(*CED*) between every two measuring points among the *N* measuring points is calculated.
- Step 3: Initially, all measuring points self-form a partition, the number of partitions is *k* = *N*, distance matrix *D* ^(1)^ between partitions is built, and the *i*th partition is $G_{i} = \left\{ {X_{(i)} } \right\}(i = 1,2, \ldots ,N)$.
- Step 4: According to the principle of the minimum sum of the squares of deviations, two partitions with the minimum comprehensive distance are combined as a new partition, the comprehensive distance *d*_*ij*_(*CED*) between the new partition and other partitions is calculated, a new distance matrix is obtained, and Steps 4 and 5 are repeated until the partitioning ends.
- Step 5: The optimal partition combination of the measuring points is obtained based on the threshold value method. Thereafter, the optimal number *K* of partitions is obtained.

### Temperature entropy

Previous studies have reported that the temperature field changes gradually, whereas the concrete structure has an obvious nonlinear self-organization mechanism and a multiscale coupling effect during the operation of concrete dams (Andrie and Chen 1975; Xie 2004; Bai 2008). The macro-mechanical properties of the concrete structure have a collaborative self-organized phenomenological response of internal multiscale physical quantities to various effects. Currently, the multiscale synergic evolution of a system is difficult to elaborate because of the limited theoretical basis and poor calculation methods. Although the system has different evolution equations at different scales and levels, energy is a general physical quantity that could run across scales and levels. To date, some researchers have studied system evolution from the perspective of energy. Reference analyzed the macro physical significance of entropy based on the available energy of closed thermal systems (Yu 1995). Reference discussed the variation law of deformation energy and the mechanism of sudden energy changes during the gradual evolution of concrete dams (Wu and Guo 2010). Reference studied the analytical methods of multi-objective decision-making during concrete dam operation based on the entropy weight (Maken et al. 2014). The abovementioned studies imply that the orderly evolution of systems can be effectively studied from the perspective of energy.

#### Entropy features of the spatial temperature field

Entropy is a basic concept in thermodynamics that is used to express a state function of a system. This concept was introduced by Rudolf Clausius in the 1850s while discussing the Carnot cycle.

9 $$dS = \left( {\frac{\delta Q}{T}} \right)_{rev}$$

where *dS* is the differential of entropy of the state function, *δQ* represents the heat exchanged between the system and environment, *rev* indicates that the process is reversible, and *T* is the temperature of the heat source. Hence, entropy is the state function of a system. Currently, the physical significance of entropy is mostly based on the microscopic expression of entropy, which was proposed by Ludwing Boltzmann in the 1870s.

10 $$S = k\ln \Omega$$

where *k* is the Boltzmann’s constant and Ω is the amount of microscopic quantum states, that is, the probability of occurrence of macroscopic state. Boltzmann’s definition states that the microscopic physical significance of entropy can be expressed as follows: entropy is system uncertainty and a measurement of the system disorder degree. According to entropy theory, when a spatial temperature field is in a more dangerous state, the system is in greater disorder and its entropy value is smaller.

#### Methods of characterizing contributions of single observation point

**Fig. 2:**
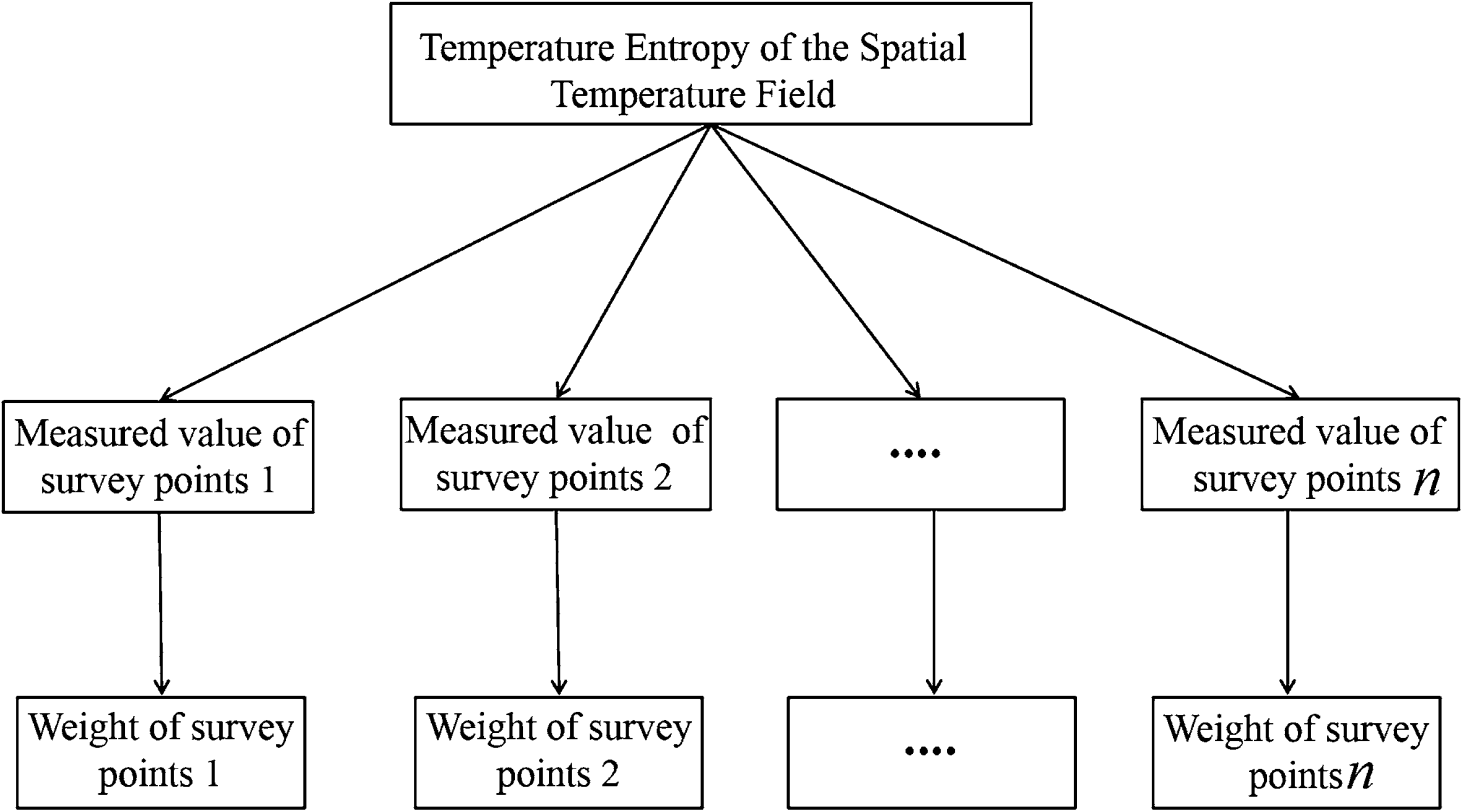
Hierarchical structure of temperature entropy of the spatial temperature field

According to principle of synergetics, if the temperature monitoring points of high concrete dams are used as the characteristic points of system analysis, the whole temperature field can be expressed by evolution equations of all characteristic points. Because synergetics theory mainly studies that how to use system’s own internal synergy to generate the ordered structure spontaneously in time, space and function. We can see that the value of single temperature monitoring points is out-of-order. However, if we take all of the temperature monitoring points as a whole, we can discover there similarity and relevance. This paper gave a quantitative description to the overall temperature of dam based on entropy structure. This is named as “temperature entropy”. Temperature entropy is a comprehensive index that integrates the effects of various factors. This index can be used to describe degree of order of the whole temperature field. The hierarchical structure of temperature entropy of the spatial temperature field is shown in Fig. 2.

#### Constructing temperature entropy of single measured value

The degree of order for a single temperature monitoring point, a dimensionless standardized value, is introduced before calculating the single-point temperature entropy. This value measures the degree of temperature order at single point. During the operation period, a concrete dam body is mainly influenced by the dead load and temperature load; consequently, the temperature variation can be viewed as a probability event (Wu 2003). For those complicated mechanical properties caused by materials, load, and environment and so on, they are reflected comprehensively in the measured temperature data from thermometers placed in the different part of dam. Therefore, when we proposed the temperature entropy expression that can describe degree of order of the temperature field in concrete dams, we didn’t consider various mechanical and thermal properties. The small probability method for calculating the monitoring index discloses that the smaller the probability of temperature reoccurrence is, the more dangerous the dam will be. Therefore, the probability of temperature occurrence at monitoring points represents the safety degree of the temperature load and can be used as an important index to measure the degree of order of the temperature field.

Here, the degree of order of the measured temperature at each monitoring point can be defined. Generally speaking, the increasing dam temperature is viewed as a positive change. Therefore, the degree of order of the *j*th measured temperature at the *i*th monitoring point (*μ*_*ij*_) can be defined as follows:

1. When temperature increases, *u*_*ij*_ is
  11 $$u_{ij} = F(x_{ij} ) = \int_{ - \infty }^{{x_{ij} }} {f_{i} (\delta )} d\delta$$

2. When temperature decreases, *u*_*ij*_ is
  12 $$u_{ij} = 1 - F(x_{ij} ) = \int_{{x_{ij} }}^{ + \infty } {f_{i} (\delta )} d\delta$$

where *f*_*i*_(*δ*) is the probability density function of the measured temperature at the *i*th monitoring point and *F*(*x*) is the corresponding probability distribution of *x*.

Formulas (11) and (12) state that larger deviations of the measured temperature of a concrete dam from the initial temperature would lead to larger *u*_*ij*_ values; otherwise, the *u*_*ij*_ will be smaller.

According to the definition of information entropy, the temperature entropy of *x*_*ij*_ can be defined as:

13 $$S_{i}^{j} = - \left[u_{ij} \ln u_{ij} + (1 - u_{ij} )\ln (1 - u_{ij} )\right] = - \sum\limits_{k = 1}^{2} {u_{ij}^{k} } \ln u_{ij}^{k}$$

where $u_{ij}^{1} = u_{ij}$ and $u_{ij}^{2} = 1 - u_{ij}$ are degree of order and degree of disorder of the monitoring point, respectively.

Obviously, Formulas (11) and (12) indicate that $0 < u_{ij}^{1} < 1$ and $0 < u_{ij}^{2} < 1$. Meanwhile, $u_{ij}^{1} + u_{ij}^{2} = 1$, which implies that the sum of the degree of order and the degree of disorder is 1. The degree of disorder decreases while the degree of order increases, and vice versa.

#### Weight entropy

The overall temperature entropy includes two levels: overall temperature entropy at the top and single-point temperature entropy at the bottom. The monitoring points interact with each other and influence the evolution of overall temperature entropy together.

Let $\{ \omega_{i} \left| i \right. = 1,2, \cdots ,n\}$(*n* is the number of total monitoring points) represent weight distribution of all temperature monitoring points. Apparently, $\omega_{i}$ is non-negative ($\omega_{i} \ge 0$). Meanwhile, the weight index meets the normalization conditions: $\sum\nolimits_{i = 1}^{n} {\omega_{i} } = 1$. Therefore, the weight distribution entropy of all monitoring points is:

14 $$S_{\omega } = - \sum\limits_{i = 1}^{n} {\omega_{i} \ln \omega_{i} }$$

The temperature monitoring point with greater contributions to the overall temperature entropy possesses the higher weight. According to the principle of synergetics, the synergic evolution equation involves both stable and unstable modals. Structural evolution is mainly determined by unstable modals. Therefore, only unstable modes dominate the orderly variation of the dam temperature when the overall temperature field change is under critical phase change. The stable modals slightly influence the orderly variation of the dam temperature.

#### Weight optimization based on projection pursuit

Given that weight can be viewed as contributions of different models to the overall system evolution, weight calculation has a very important role in analysis of temperature entropy. In order to determine the weight distribution of monitoring points, this paper explored the implied information from the spatial temperature field data of a dam by projection pursuit analysis (PPA) (Friedman and Turkey 1974; Flick et al. 1990; Kumar et al. 2008). As a high-dimensional data analysis method, PPA could identify data structures or characteristics in the projection space by projecting high-dimensional monitoring data into low-dimensional spaces. PPA can analyze weights of evaluation index values according to the global data distribution and local data concentrations. Data dimension reduction is the main goal of PPA. The analysis integrates *m*-dimensional sequential values of measured dam temperature ($\left\{ {y_{ij} \left| {i = 1\;\sim\;n;\, j = 1\;\sim\;m} \right.} \right\}$) into one-dimensional comprehensive projection value ($G(i)$) toward the projection direction of *P*:

15 $$G(i) = \sum\limits_{j = 1}^{m} {p_{j} y_{ij} } ,\quad (i = 1,2, \ldots ,n)$$

where *P* is the unit length–width vector, $P = \{ p_{1} ,p_{2} , \ldots ,p_{m} \}$.

Given the standard sample set of the measured dam temperature, *P* determines the form of the projection index function (*Q*(*P*)). Consequently, *Q*(*P*) only changes while *P* changes. Therefore, the optimum projection direction can be estimated by calculating the maximum *Q*(*P*):

16 $${\rm M}ax:H(p) = S_{G} \cdot Q_{G}$$

Meanwhile, *P* must satisfy the following constraint:

17 $$\sum\limits_{j = 1}^{m} {p^{2} (j) = 1}$$

where $S_{G} = \left[ {\sum\nolimits_{i = 1}^{n} {(G(i) - \bar{g}(i))^{2} /(n - 1)} } \right]^{0.5}$ is the dispersity of projections; $Q_{G} = \sum _{i = 1}^{n} \sum_{j = 1}^{n} \left( {R - r_{ij} } \right) \cdot f\left( {R - r_{ij} } \right)$ is a projection index function, which represents the local density of one-dimensional data points along *P*; $\bar{g}(i)$ is the mean value of sequence {*G*(*i*), *i* = 1, 2, …, *n*}; *R* is the window radius of local density, which is calculated as 0.1 *S*_*G*_ in this paper; *r*_*ij*_ is the distance between projections (*r*_*ij*_ = |*G*(*i*) − *G*(*j*)|); *f*(*t*) is a unit step function. When *t* ≥ 0, *f*(*t*) = 1; when *t* < 0, *f*(*t*) = 0.

By substituting the optimum P* into Formula (15), the projection of sample point could be gained. Subsequently, the projection is normalized to obtain the weights of the deformation monitoring points:

18 $$\omega_{i} = \frac{{G^{*} (i)}}{{\sum\nolimits_{j = 1}^{n} {G^{*} (j)} }},\quad (i = 1,2, \ldots n)$$

where *G* ^*^(*i*) is the optimum projection of the *i*th evaluation index and *ω*_*i*_ is the weight of the *i*th evaluation index.

#### Construction of the temperature entropy of the spatial temperature field

The degree of order sequence ($\{ u_{ij}^{1} \}$), the degree of disorder sequence ($\{ u_{ij}^{2} \}$), the deformation entropy sequence ($\{ S_{i}^{j} \}$), and the weight distribution entropy of the spatial temperature field were calculated by using Formula (11)–(14). Based on these equations, the formula to calculate the multi-point temperature entropy was deduced as follows. The degree of order and the weights of the monitoring points were combined. For the *i*th monitoring point, the contribution of its degree of order to the overall temperature entropy is $\omega_{i} u_{ij}^{1}$, whereas the contribution of its degree of disorder to the overall deformation entropy is $\omega_{i} u_{ij}^{2}$. According to definition of generalized information entropy, the orderly entropy of the whole temperature field is:

Formula (19) can be rewritten as:

19 $$S^{j} = - \sum\limits_{i = 1}^{n} {\sum\limits_{k = 1}^{2} {\omega_{i} u_{ij}^{k} (\ln\, \omega_{i} + \ln u_{ij}^{k}} } ) = - \sum\limits_{i = 1}^{n} {\sum\limits_{k = 1}^{2} {\omega_{i} u_{ij}^{k} \ln \omega_{i} - \sum\limits_{i = 1}^{n} {\sum\limits_{k = 1}^{2} {\omega_{i} u_{ij}^{k} \ln u_{ij}^{k}} } } }$$

Two items in Formula (19) can be simplified into:

20 $$- \sum\limits_{i = 1}^{n} {\sum\limits_{k = 1}^{2} {\omega_{i} u_{ij}^{k} \ln \omega_{i} } } = - \sum\limits_{i = 1}^{n} {\omega_{i} \ln \omega_{i}} \sum\limits_{k = 1}^{2} {u_{ij}^{k} } = - \sum\limits_{i = 1}^{n} {\omega_{i} \ln \omega_{i}} = S_{\omega }^{i}$$

21 $$- \sum\limits_{i = 1}^{n} {\sum\limits_{k = 1}^{2} {\omega_{i} u_{ij}^{k} \ln u_{ij}^{k} } } = - \sum\limits_{i = 1}^{n} {\omega_{i} \sum\limits_{k = 1}^{2} {u_{ij}^{k} } } \ln u_{ij}^{k} = \sum\limits_{i = 1}^{n} {\omega_{i} S_{i}^{j} }$$

By substituting Formulas (20) and (21) into Formula (19), and the overall temperature entropy can be simplified into:

22 $$S^{j} = S_{\omega }^{j} + \sum\limits_{i = 1}^{n} {\omega_{i} S_{i}^{j} }$$

Formula (22) reveals that the overall temperature entropy involves the weight distribution entropy ($S_{\omega }^{j}$) and the weighted average of the temperature entropy of different monitoring points ($\sum\nolimits_{i = 1}^{n} {\omega_{i} S_{i}^{j} }$).

### Establishment of an early-warning index based on the temperature entropy

The measured temperature data of concrete dams collected from each survey were transformed through Formula (22) to obtain the sequential value of temperature entropy ({*S*_*i*_}). Combined with practical situations of dams, the most unfavorable temperature entropy for the load combination (*S*_*mi*_) was chosen. *S*_*mi*_ is a random variable. A sample space with *N* samples can be gained from monitoring sequence:

23 $$X = \{ S_{m1} ,S_{m2} , \ldots ,S_{mn} \}$$

Subsequently, the distribution of this sample space was tested by small-sample statistical testing methods (e.g., the A–D method or the K–S method) to determine the distribution function *F*(*X*) of its probability density *f*(*x*). K–S (Kolmogorov–Smirnov) method bases on empirical distribution function, which is used to determine whether a sample is from a specific distribution. A–D (Anderson–Darling) method is a correction of A–D method and gives weight to the distribution of the tail. Besides, K–S inspection has nothing to do with the specific distribution, that’s to say, its critical value doesn’t depend to the tested specific distribution.

*S*_*m*_ is defined as the extreme value of the temperature entropy (Jaynes 1963). When *S* ≤ *S*_*m*_, the dam will show abnormalities or danger. The failure probability of the dam is:

24 $$P(S \le S_{m} ) = P_{\alpha } = \int_{ - \infty }^{{S_{m} }} {f(x)dx}$$

After the distribution of deformation entropy (*S*) was calculated, most attention focused on the determination of the failure probability (*P*_*α*_, herein referred to as *α*) to estimate *S*_*m*_. Based on *α* and *f*(*x*), the early-warning index of temperature entropy can be determined:

25 $$S_{m} = F^{ - 1} (\overline{S} ,\sigma_{S} ,\alpha )$$

**Fig. 3:**
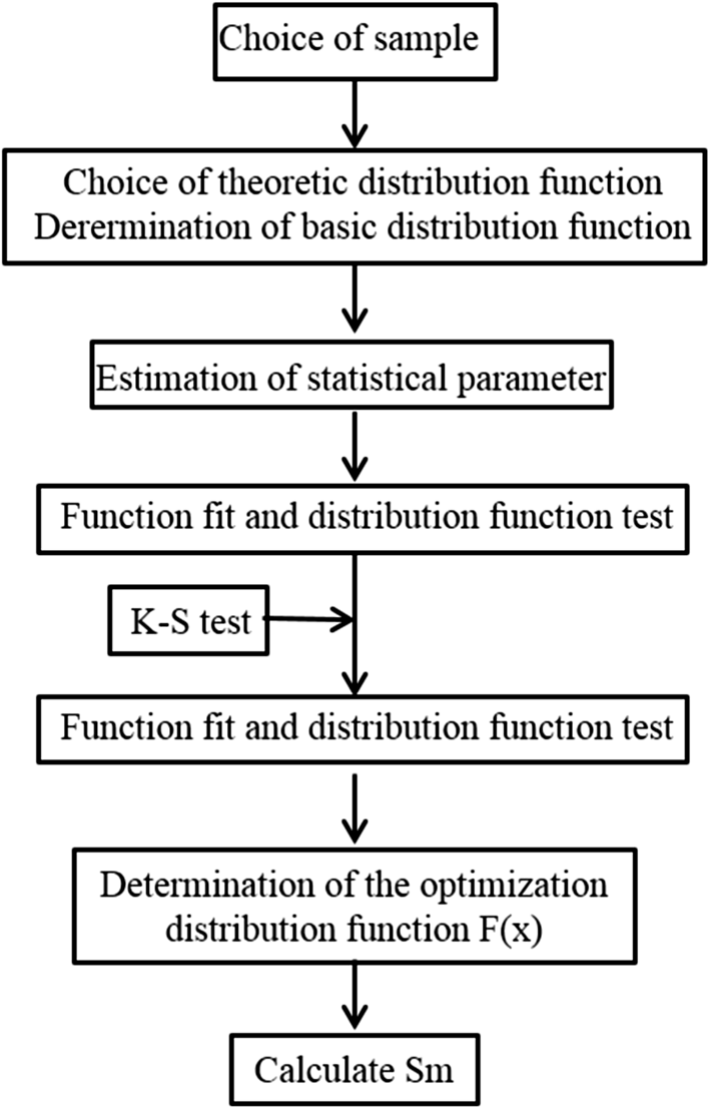
Flow diagram of the calculation of spatial temperature entropy

The spatial temperature entropy index (*S*_*m*_) can be determined as soon as the optimum fitting distribution function was identified (Fig. 3).

*S*_*m*_ is the early-warning index value under the failure probability of *α*. Dam security can be evaluated by comparing the temperature entropy and *S*_*m*_. If temperature entropy is smaller or equal to *S*_*m*_, attention should be paid to determine probable causes, to strengthen the monitoring system, and to analyze whether other monitoring items have abnormalities. An appropriate *α* should be chosen to calculate *S*_*m*_. The value of *α* is determined by various factors, such as engineering grade, engineering scale, operation progress and so on. Meanwhile, several confidence values of *α* shall be set for the dam risk management: *α*_1_, *α*_2_, …, *α*_*n*_(*α*_1_ > *α*_2_ > ···*α*_*n*_).The corresponding *S*_*m*_ ($S_{{m_{1} }} ,S_{{m_{2} }} , \ldots ,S_{{m_{n} }}$) should be calculated to get the multi-level early warning index of the overall temperature field in dams.

## Result

### Project overview

**Fig. 4:**
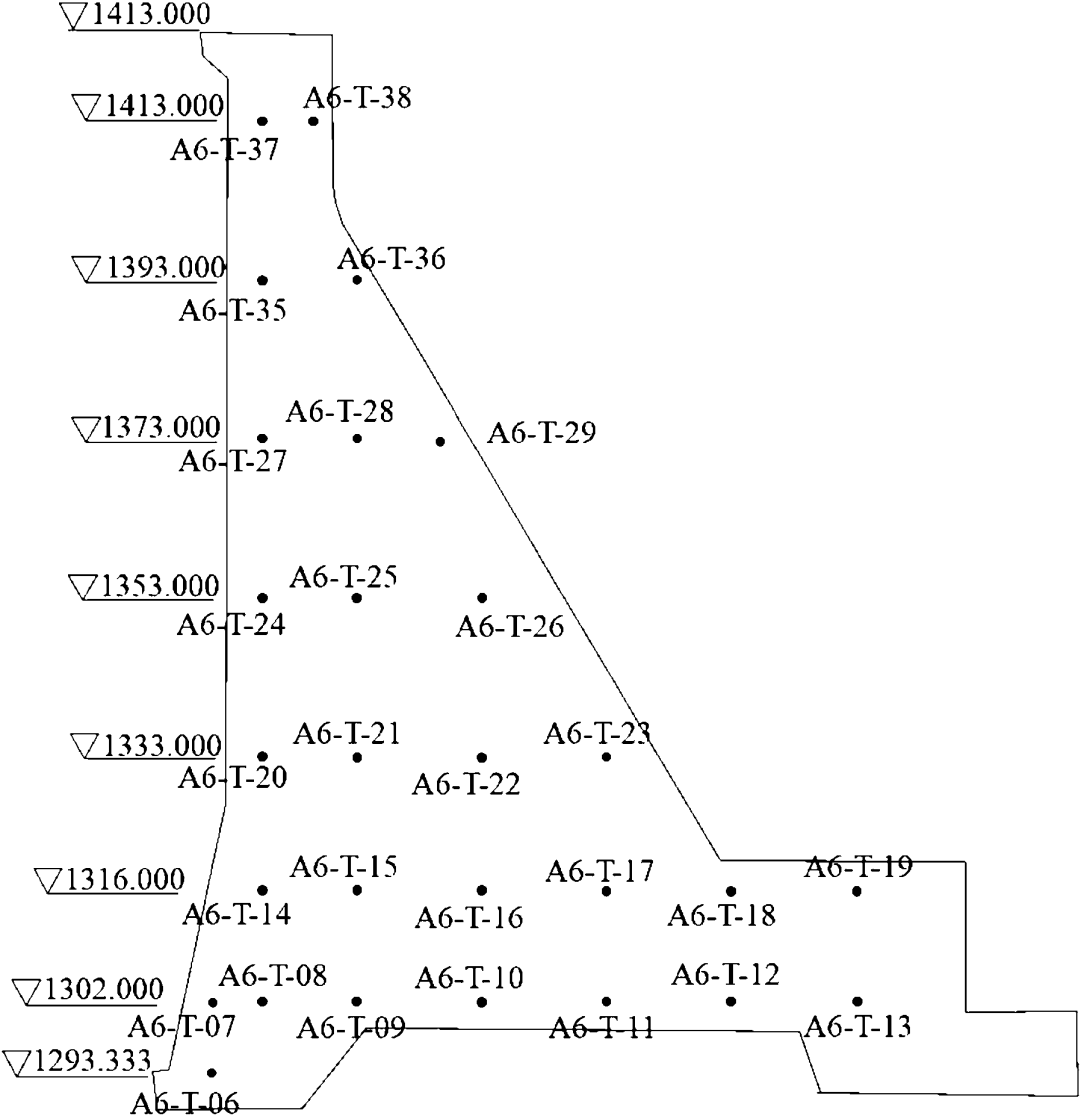
Thermometer distribution of the 6th dam block

The studied high concrete dam is mainly composed of the roller compacted concrete gravity dam, the exterior overflow dam section, the discharge chute and stilling pool, the underport at right bank for flood discharge (sand flushing), the underport at left bank for sand flushing, the permanent buildings (e.g. dam-rear power plant and access tunnel), and the temporary buildings (e.g. diversion tunnel and cofferdam). The elevation at the top is 1424 m. The biggest height is 160 m and the top length is 640 m. This paper mainly studied the 6th dam block. The thermometer distribution is shown in Fig. 4.

To study the temperature field changes during dam operation and the safe impounding period, temperature monitoring data from August 1, 2007 to January 20, 2014 were used as the sample data in this paper. Temperature data of all monitoring points within the 6th dam block were used to calculate temperature entropy and early-warning index value.

### Partitioning calculation results

**Fig. 5:**
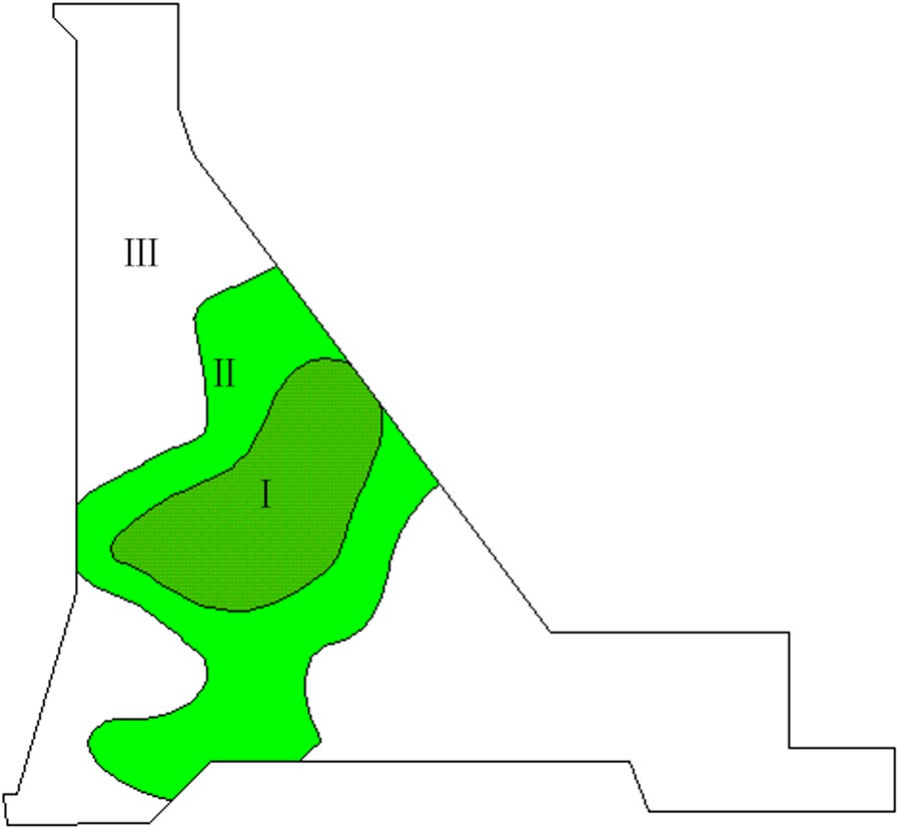
Partition map of the spatial temperature field of the 6th dam block

All measuring points were finally partitioned into three major categories, and the dam was divided into three regions according to the locations of the measuring points. Variation trends and rules of the measuring points within the categories obtained through clustering were identical. Each category of the measuring points could comprehensively describe the features of the spatial temperature field. A temperature field partition map of the 6th dam block is illustrated in Fig. 5. Table 1 provides the measuring point clustering partition table of the spatial temperature field.

**Table 1:** Measuring point clustering partition table of the spatial temperature field

Partitions	Measuring points											
Partition I	A6-T-15	A6-T-16	A6-T-21	A6-T-22	A6-T-26							
Partition II	A6-T-09	A6-T-10	A6-T-15	A6-T-16	A6-T-20	A6-T-23	A6-T-25	A6-T-29				
Partition III	A6-T-06	A6-T-07	A6-T-08	A6-T-11	A6-T-12	A6-T-13	A6-T-14	A6-T-17	A6-T-18	A6-T-19	A6-T-24	A6-T-27
	A6-T-28	A6-T-35	A6-T-36	A6-T-37	A6-T-38							

### Calculation results of the weight *ω*_*i*_

**Fig. 6:**
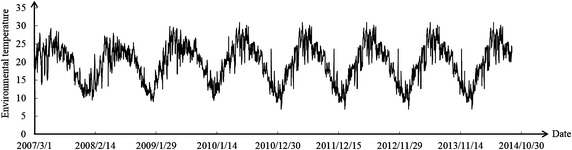
Curve of environmental temperature

**Fig. 7:**
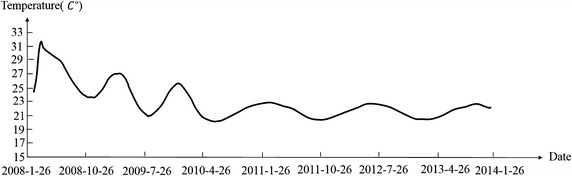
Temperature monitoring sequence of A6-T-21

Figures 6 and 7 reveal that the environmental temperature obviously influenced the dam body’s temperature. Temperature change was divided into the stage of temperature rise and stage of temperature drop. The weight of deformation at observation point in two stages was calculated. The results are shown in the Table 2.

**Table 2:** Weight table of the observation points of the 6th dam block in the rising and declining air temperature phases

Partitions	Measuring points	Temperature rise phase	Temperature decline phase
Partition I	A6-T-15	0.13	0.23
	A6-T-16	0.14	0.09
	A6-T-21	0.34	0.39
	A6-T-22	0.32	0.15
	A6-T-26	0.07	0.14
Partition II	A6-T-09	0.07	0.05
	A6-T-10	0.09	0.11
	A6-T-15	0.12	0.14
	A6-T-16	0.13	0.11
	A6-T-20	0.26	0.27
	A6-T-23	0.18	0.14
	A6-T-25	0.04	0.05
	A6-T-29	0.06	0.07
Partition III	A6-T-06	0.06	0.04
	A6-T-07	0.04	0.07
	A6-T-08	0.03	0.07
	A6-T-11	0.06	0.05
	A6-T-12	0.04	0.05
	A6-T-13	0.05	0.04
	A6-T-14	0.07	0.08
	A6-T-17	0.06	0.06
	A6-T-18	0.08	0.04
	A6-T-19	0.09	0.08
	A6-T-24	0.06	0.07
	A6-T-27	0.07	0.05
	A6-T-28	0.03	0.07
	A6-T-35	0.06	0.04
	A6-T-36	0.05	0.05
	A6-T-37	0.07	0.06
	A6-T-38	0.08	0.08

### Calculation of spatial temperature entropy

**Fig. 8:**
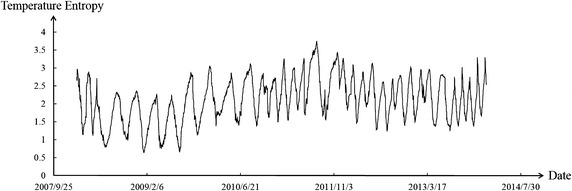
Temperature entropy curve of partition I

**Fig. 9:**
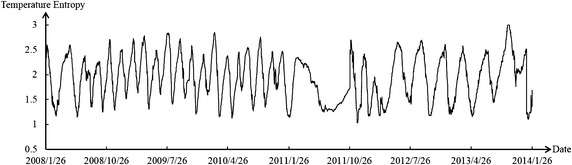
Temperature entropy curve of partition II

**Fig. 10:**
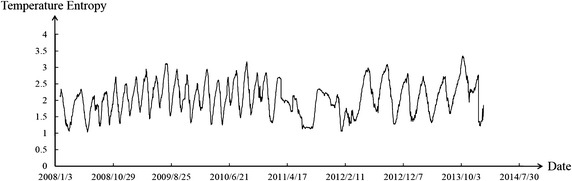
Temperature entropy curve of partition III

Figures 8, 9, 10 show the temperature entropy curve of all partitions. Table 3 shows the annual extreme temperature entropies of all partitions.

**Table 3:** Annual extreme temperature entropies

Temperature entropy	Year					
	2008	2009	2010	2011	2012	2013
Minimum value of partition I	0.763	0.632	1.235	1.032	1.124	1.342
Minimum value of partition II	1.142	1.208	1.126	1.034	1.171	1.164
Minimum value of partition III	1.132	1.418	1.324	1.053	1.132	1.145

### Calculation of early-warning index of concrete dam

In this paper, two-stage warning indicators were set according to the practical running of this project and danger: *α* = 5% is the primary warning that is mainly used to discriminate and handle early dangerous case, whereas *α* = 1% is the secondary warning that is mainly used to determine grave danger and prevent urgent danger. Table 4 shows the K–S test results of all partitions. Table 5 shows the parameter values of the probability density function of all partitions. Table 6 shows the early-warning index values of temperature entropy of all partitions.

**Table 4:** K–S test results

Probability distribution	Partition I	Partition II	Partition III
Lognormal distribution	0.32	0.17	0.25
Normal distribution	0.41	0.21	0.31
Uniform distribution	0.78	0.95	0.86
Triangular distribution	0.32	0.44	0.43
Exponential distribution	0.44	0.46	0.65
*γ* distribution	0.34	0.53	0.21
*β* distribution	0.53	0.71	0.53
The most reasonable probability distribution	Normal distribution	Normal distribution	Normal distribution

K–S test shows that the annual extreme temperature entropies of all partitions satisfy the normal distribution

**Table 5:** Parameter values of the probability density function

Partition	Parameter values	
	*μ*	*σ* ^2^
Partition I	1.021333	0.07549
Partition II	1.140833	0.003519
Partition III	1.200667	0.019356

**Table 6:** Early-warning index values of all partitions

Early-warning level	*α* = 5%	*α* = 1%
Early-warning index values		
PartitionI	0.569363	0.381156
Partition II	1.04325	1.002615
Partition III	0.971805	0.876503

In this paper, the early-warning index values of all partitions was analyzed through the theoretical method. If the temperature entropy value of partition I reaches 0.569363, the dam is in the state of primary warning. If the temperature entropy value of partition I reaches 0.381156, the dam is in the state of secondary warning. If the temperature entropy value of partition II reaches 1.04325, the dam is in the state of primary warning. If the temperature entropy value of partition II reaches 1.002615, the dam is in the state of secondary warning. If the temperature entropy value of partition III reaches 0.971805, the dam is in the state of primary warning. If the temperature entropy value of partition III reaches 0.876503, the dam is in the state of secondary warning.

## Conclusions

This study proposed a scientific warning theory on global behavior of the temperature field and verified the proposed method through an engineering project. Accordingly, the following conclusions are drawn.

1. Based on the Ward spatial clustering, partitioning of the spatial temperature field is conducted according to the intimacy degree among all the observed values. The partitioning principle attempts to make the change rule similarity degree within partitions as high as possible while make the change rule similarity between partitions as low as possible.
2. The degree of order and degree of disorder of the temperature monitoring points were defined by the probability method. The weight of each temperature monitoring points was explored via projection pursuit.
3. According to the coordinated and orderly evolution characteristics of the spatial temperature field, a temperature entropy expression that can describe degree of order of the spatial temperature field in concrete dams was established.
4. The early-warning index of temperature entropy was set up via the small probability method according to the calculated sequential value of temperature entropy.
